# *SERPINC1* mutations and thrombotic events in inherited antithrombin deficiency: a study on the han population of East China

**DOI:** 10.1186/s13023-026-04200-0

**Published:** 2026-03-26

**Authors:** Fei Xu, Xiaoli Chen, Qiyu Xu, Anqing Zou, Xiaolong Li, Mingshan Wang, Lihong Yang, Haixiao Xie

**Affiliations:** 1 Department of Clinical Laboratory, Key Laboratory of Clinical Laboratory Diagnosis and Translational Research of Zhejiang Province, The First Affiliated Hospital of Wenzhou Medical University, Wenzhou, Zhejiang China; 2 Department of Neurology, The First Affiliated Hospital of Wenzhou Medical University, Shangcai Village, Ouhai District, Wenzhou, Zhejiang China

**Keywords:** Antithrombin deficiency, *SERPINC1* gene, Mutation, Thrombophilia, In silico bioinformatic tools

## Abstract

**Background:**

Inherited antithrombin deficiency (ATD), a rare autosomal dominant disorder due to *SERPINC1* gene mutations, is the most severe inherited thrombophilia. Limited literature exists that focuses on ATD and its mutations in the Chinese population. This study aimed to characterize *SERPINC1* gene mutations in a Chinese cohort and to explore their relationship with thrombophilia.

**Methods:**

Coagulation screening results and clinical data were meticulously collected from 23 unrelated probands with ATD and their family members. Genomic DNA was extracted and subjected to PCR amplification and direct sequencing. Putative mutations were analyzed using in silico bioinformatic tools. Mutant antithrombin (AT) proteins were expressed in HEK293 cells, and ELISA was used to detect wild-type and mutant AT. RT-qPCR was used to measure AT mRNA expression in transfected cells.

**Results:**

Among the 23 probands, 15 (65.2%) exhibited concurrent reductions in both AT: A and AT: Ag (type I defects), while the remaining 8 (34.8%) had normal AT: Ag levels (type II defects). Genetic analysis revealed a spectrum of 21 distinct mutations across 87.0% (20/23) of the probands. Most were point mutations predicted to be deleterious and were primarily located in exons 5 and 3. Among the 20 mutation carriers, 15 (75%) were heterozygous and most of them experienced thrombosis with identifiable triggers. The other 5 (25%) were compound heterozygous and primarily presented with spontaneous thrombosis. Notably, the missense mutations c.1346T > A and c.442T > C were recurrent. These mutations exhibited high heterogeneity, with no ethnic-specific mutations observed. In vitro expression confirmed that synthesis and/or secretion defects in the mutant proteins are the primary mechanism underlying the antithrombin deficiency.

**Conclusions:**

*SERPINC1* gene analysis benefits asymptomatic family members, especially child-bearing women, by informing venous thromboembolism prevention strategies and guiding anticoagulant choice in cases involving heparin-binding site mutations. This underscores the essential role of genetic diagnosis in ATD management.

**Supplementary Information:**

The online version contains supplementary material available at 10.1186/s13023-026-04200-0.

## Introduction

Antithrombin (AT), a glycoprotein primarily synthesized in the liver, is a key physiological anticoagulants. It has a molecular weight of 58,200 and is produced from a 464-amino acid precursor following the cleavage of a 32-amino acid signal peptide. Structurally, AT comprises three β-sheets (A to C), nine α-helices (A to I), and a reactive center loop. Functionally, AT plays a crucial role in the coagulation system by inactivating thrombin, Factor IXa, Factor Xa, Factor XIa, and Factor XIIa in both the intrinsic and extrinsic pathways to prevent pathological thrombosis. Moreover, AT exhibits anti-inflammatory, anti-angiogenic, and anti-protease kinase activities.

Inherited antithrombin deficiency (ATD), first reported by Olav Egeberg in 1965, is a rare genetic disorder with an estimated prevalence of 0.02%-0.2% in the general population, but increases to 1%-5% in patients with venous thromboembolic diseases like deep vein thrombosis (DVT) or pulmonary embolism (PE) [1, 2]. ATD is primarily caused by autosomal dominant mutations in the serpin family C member 1 (*SERPINC1*) gene [3–5], predisposing patients to a heightened risk of thromboembolism and resulting in recurrent venous and arterial thromboses [6, 7]. Quantitative (type I) and qualitative (type II) defects have been described in patients with inherited ATD [7]. In type I defects, both the level of AT antigen and its activity are reduced [8]. In contrast, type II deficiency exhibit a normal AT antigen level, but reduced AT activity due to a dysfunctional protein. Type II deficiency can be further divided into three subtypes: type IIa, resulting from mutations in the thrombin binding site; type IIb, due to mutations in the heparin binding site; and type IIc, where mutations near the reactive center loop (RCL) have pleiotropic impacts on both heparin and thrombin interactions [7].

The *SERPINC1* gene, located on chromosome 1q23-25, spans 13.4 kb and contains 7 exons and 6 introns. Exon 6 encodes RCL, which forms the active site of AT, while exon 2 encodes the majority of the heparin-binding domain. Furthermore, *SERPINC1* is highly sensitive to alterations, and even minor nucleotide variations can lead to significant structural and functional impacts, thereby promoting thrombosis [1, 9]. To date, over 500 causal variants in *SERPINC1* have been documented in the HGMD database http://www.hgmd.cf.ac.uk/ac/gene.php?gene=SERPINC1). Most are point mutations, with missense and nonsense mutations accounting for more than half of these. Notably, few polymorphisms have been found in the *SERPINC1* gene, mostly in introns [10].

To obtain detailed clinical and molecular data and given the scarcity of in vitro studies, we conducted a study on 23 AT-deficient probands from the First Affiliated Hospital of Wenzhou Medical University to investigate genotype-phenotype correlations and molecular characteristics.

## Materials and methods

### Participants

From 2013 to 2024, we retrospectively analyzed 23 unrelated ATD probands, consisting of 12 males and 11 females with an age range of 17 to 83 years. Additionally, we performed pedigree investigations for 100 relatives from 17 probands’ families. The diagnosis of ATD was confirmed through repeated low plasma AT activity measurements and a personal or family history of thromboembolic events. Clinical data, including thromboembolic events, family history, and consanguinity, were systematically gathered from the probands and their medical records.

Proband 1, a 21-year-old male with a history of DVT, presented with recurrent headaches. Computed tomography (CT) revealed thrombosis in the left transverse, sigmoid, and straight sinuses, and ultrasound confirmed DVT in the right lower extremity. Proband 2, a 27-year-old female, was admitted for a sudden puerperal headache accompanied by nausea and vomiting. Magnetic resonance venography (MRV) showed thrombosis in the straight sinus, confluence of sinuses, and left transverse sinus. Proband 3, a 36-year-old 8-week pregnant woman, was admitted to the emergency department (ED) after severe vomiting and a 5-day headache. Cranial MRI confirmed cerebral venous sinus thrombosis (CVST). Despite intervention, her condition deteriorated, and she unfortunately died. Proband 4, a 57-year-old male, presented with bilateral lower limb swelling and gait instability for half a month. Ultrasound examination showed left femoral vein thrombosis, leading to a diagnosis of left lower limb DVT. Proband 5, a 34-year-old male, reported chest tightness for 3 months and had a 15-year DVT history. Pulmonary angiography (PA) revealed multiple thrombi in both pulmonary arteries. Proband 6, a 83-year-old male with a history of hypertension and DVT, was admitted for sudden-onset chest tightness and pain that had lasted for one day. One week later, follow-up coronary angiography revealed a thrombus shadow distal to the stent. Proband 7, a 30-year-old male, was hospitalized for waist and groin pain and left lower limb swelling. Emergency CT and ultrasound detected left lower limb DVT. After showing clinical improvement with treatment, he was discharged but readmitted a week later with left chest pain and hemoptysis (4-5times/day). PA confirmed pulmonary infarction. His family history was significant for DVT (affecting his father, uncle and brother), and his deceased grandmother died of PE. Proband 8, a 24-year-old male, presented to our vascular surgery department with 3-day left lower limb pain and swelling that worsened with walking and was associated with numbness. Ultrasound confirmed left lower limb DVT with thrombi in the left iliac and femoral veins. His father had a history of right popliteal vein DVT at 35. Proband 9, a 33-year-old female, presented with over two days of sudden swelling and pain in the left lower extremity. Ultrasound showed multiple thrombi in the left external iliac, common femoral, profunda femoris veins and the great saphenous vein’s initial segment, leading to a diagnosis of left lower extremity DVT. She reported a family history of thrombosis affecting cousins on both sides. Proband 10, a 74-year-old male with a 5-year history of hypertension, suddenly developed left-sided limb weakness, which resulted in a fall. Ultrasound revealed thrombi in the intermuscular venous plexus of both legs. Proband 11, a 30-year-old 26 weeks pregnant woman, was hospitalized for two days of left lower-limb pain. Ultrasound confirmed left iliac vein thrombosis. Four years ago, she had a cesarean section followed by PE. She reported a strongly positive family history of thrombosis: her mother had PE, her maternal uncle had intestinal vascular thrombosis, and her maternal grandfather died of PE at the age of 64. Proband 12, a 24-year-old female with three spontaneous abortions, was found to have reduced AT activity during infertility evaluatio. She reported no personal thrombosis but a significant family history (paternal death from VTE at age 53). Proband 13, a 32-year-old female, presented to our hospital’s reproductive center due to three spontaneous abortions. At the age of 25, she had developed a left lower limb DVT during bed rest for pregnancy preservation. Proband 14, a 74-year-old male, presented to our ED with a 2-day history of sudden swelling, pain, and weakness in both lower limbs. Ultrasound confirmed bilateral external iliac vein thrombosis and DVT in both lower limbs. Proband 15, a 32-year-old female, was found to have reduced AT activity and embryonic arrest during a 6-week prenatal screening. Proband 16, a 25-year-old male, presented with 4-day history of left lower limb swelling. Ultrasonography showed bilateral external iliac vein thrombosis and DVT in both lower limbs. PA revealed embolisms in the left lower pulmonary artery trunk and multiple pulmonary arteries of the left lung’s upper and lower lobes. Proband 17, a 53-year-old female with a history of bilateral lower-limb DVT and left muscular vein thrombosis, was admitted to our ED for abdominal pain persisting for over one month. The CT scan showed superior mesenteric vein thrombosis and associated intestinal necrosis. Proband 18, a 37-year-old male with a history of PE five years earlier, presented with a 10-day history of right lower limb swelling and pain, accompanied by 1-day of dyspnea. PA showed scattered thrombi in both lungs. He reported a strong family history of thrombosis, affecting his eldest and second maternal uncle, as well as his eldest maternal aunt. Proband 19, a 63-year-old male, presented with progressive pain and numbness in the left lower extremity. Ultrasound confirmed left iliac and femoral vein thrombosis. Proband 20, a 24-year-old male, was admitted to our hospital with an 8-hour history of chest tightness, weakness, unsteady walking, and left thigh swelling. Ultrasound showed left iliac and femoral vein thrombosis. PA indicated pulmonary embolism. Proband 21, a 17-year-old girl, suffered multiple fractures from a high-altitude fall. While the admission ultrasound was negative for thrombosis, a follow-up study 20 days later detected thrombi in the left lower-limb deep veins. Proband 22, a 28-year-old pregnant woman (11-weeks), presented with a 15-day history of left buttock pain and a 10-day history of chest tightness. PA indicated a left pulmonary artery embolism. Proband 23, a 69-year-old female, presented with 3-day history of lower back pain following trauma. PA showed extensive pulmonary embolism, and ultrasound detected thrombosis in the left lower-limb varicose veins, right intermuscular veins, and bilateral lower-leg intermuscular varicose veins. Individuals with acquired ATD or a history of liver or kidney disease were excluded from the study. The phenotype and genotype data of the probands and their relatives are summarized in Supplementary Table 1.

### Blood samples

Peripheral venous blood (2.7mL) was collected from each of 123 individuals suspected of having inherited AT deficiency using BD Vacutainer^®^ tubes with buffered 0.129 M sodium citrate. Platelet-poor plasma (PPP) was isolated by centrifugation at 3000 rpm for 15 min at 8℃ for subsequent coagulation assays. The lower layer was then used for genomic DNA extraction.

### Coagulation assays

Plasma samples from all available subjects were assayed according to standard methods in the coagulation laboratory of the first affiliated hospital of Wenzhou Medical University. All activity measurements were performed on a Stago STA-Max analyzer (Diagnostica Stago, Asnieres sur Seine, France) following the manufacturer’s instructions, plasma protein C (PC: A) and antithrombin (AT: A) activities were measured by the chromogenic substrate method, while protein S (PS: A) was determined by the clotting method. .Antithrombin antigen (AT: Ag) was quantified with an ELISA kit (Jianglai, China) following the manufacturer’s protocol. A total of 150 healthy subjects were recruited as controls for this study. They consisted of 78 males and 72 females, with an average age of 34 years (range:19–62 years). None of them had a history of abnormal bleeding or thrombotic tendencies, nor did they have liver or kidney disease. All participants provided written informed consent.

### Mutation analysis of AT-deficient families

To confirm AT mutations, genomic DNA was extracted from citrate-treated leukocytes using the TIANamp Genomic DNA Kit (TIANGEN, Beijing, China) as per the manufacturer’s instructions. The coding regions of the *SERPINC1* gene, along with intron-exon boundaries, were amplified by PCR with intronic primers. The PCR products were then analyzed via 1.2% agarose gel electrophoresis. Positive products were purified and sent to Personal Gene Technology Co., Ltd. (Shanghai, China) for direct sequencing. Sequencing data were evaluated against the reference *SERPINC1* sequence from NCBI GenBank (NC_000001.11) using Chromas software. Sequence variants were described according to HGVS guidelines.

### Screening for other common thrombophilia gene mutations

We assessed additional thrombosis risk factors in probands and their family members, including prothrombin G20210A mutation, factor V Leiden mutation, and antiphospholipid antibodies.

### In silico analysis

We used ClustalX-2.1-win software (HomoloGene, http://www.ncbi.nlm.nih.gov/homologene) to perform a multiple sequence alignment and assess the conservation across Homo sapiens and related species. To assess the potential disease causality of identified mutations, we employed the following bioinformatic tools: PolyPhen-2 (http://genetics.bwh.harvard.edu/pph2/), PROVEAN (http://provean.jcvi.org/seq_submit.php), MutationTaster (http://www.mutationtaster.org), Franklin (https://franklin.genoox.com/) and SIFT (http://sift.jcvi.org/). In addition, we constructed a molecular model of AT based on its crystal structure (PDB, http://www.rcsb.org, ID: 1ANT) and used PyMol (http://www.pymol.org) and Swiss-Pdb Viewer (https://spdbv.unil.ch) to visualize the mutations.

### In vitro expression

For in vitro gene overexpression studies using the pCDH-copGFP-T2A-Puro plasmid vector, the wild-type antithrombin (AT-WT) plasmid and the empty vector control were purchased from Nanjing GenScript Biotech Co., Ltd. The corresponding expression plasmids were constructed using the wild-type AT plasmid as a template, according to the protocol of the QuikChange Lightning Site-Directed Mutagenesis Kit. Mutagenic primers were designed with the PrimerX online tool (http://www.bioinformatics.org/primerx/) and synthesized by Nanjing GenScript Biotech Co., Ltd. The plasmids were purified using agarose gel electrophoresis. HEK293T cells were cultured in DMEM supplemented with 10% FBS at 37℃ under 5% CO₂ and were transfected with recombinant AT expression vectors using Lipofectamine^®^ 3000.

After 48 h of transfection, total RNA was extracted using TRIzol™ Reagent and reverse-transcribed into cDNA with HiScript II Q RT SuperMix for qPCR. The mRNA expression level of *SERPINC1* was analyzed by RT-qPCR using ChamQ Universal SYBR qPCR Master Mix on a Bio-Rad CFX96 system (Bio-Rad, USA), with GAPDH serving as the endogenous control. All reagents for reverse transcription and qPCR were obtained from Vazyme Biotech Co., Ltd (Nanjing, China). Concurrently, AT: Ag levels in both the cell culture supernatant and lysate were quantified using a commercial Human Antithrombin-III ELISA Kit (Jianglai, China) according to the manufacturer’s instructions.

## Results

### Coagulation assays

The AT: A levels in all probands were mainly in the range of 40–60%. Among them, 15 cases (65.2%) exhibited a simultaneous reduction in AT: Ag and thus were classified as type I defects, while the remaining 8 cases (34.8%) with normal AT: Ag levels were categorized as type II defects. Relatives with the same mutations as the probands showed highly consistent laboratory test results, while those without mutations had normal findings. Thrombophilia screening of all individuals found no significant abnormalities in Protein C activity, Protein S activity, or anti-cardiolipin antibody combinations. The details of the 23 probands are summarized in Table 1, while the results of monitoring these 23 families are presented in Supplementary Table 1.

**Table 1:** Laboratory and clinical data of 23 probands with inherited antithrombin deficiency

*P*	G/A	Antithrombin analysis				Nucleotide change	Protein change	Genotype	Type	T	MTh	RT	Onset age	Trigger	Antithrombotic therapy	C/F	FHT
		PC: A(%)	PS: A(%)	AT: A(%)	AT: Ag(mg/L)												
1	M/21	112	101	43	113	c.318_319insT^Δ^ c.922G > T^Δ^	p.Asn75* p.Gly276Cys	Comp.Het	Ⅰ	YES	YES	YES	21	NO	PIVT→IV Heparin →Rivaroxaban	7/9	YES
2	F/27	119	110	49	52	c.1358T > C	p.Ile421Thr	Het.	Ⅰ	YES	NO	NO	27	Preg	Subcutaneous LMWH→Dabigatran	3/7	NO
3	F/36	71	89	35	45	NA	NA	NA	Ⅰ	YES	NO	NO	36	Preg, Immobil	PIVT→IV Heparin	NA	NO
4	M/59	95	103	46	303	c.442T > C	p.Ser116Pro	Het.	Ⅱb	YES	NO	NO	59	NO	IV Heparin	NA	NO
5	M/34	89	96	66	330	c.290 A > C	p.His65Pro	Het.	Ⅱb	YES	YES	YES	19	Smoking	Subcutaneous LMWH→Warfarin	NA	NO
6	M/83	81	97	28	298	c.235 C > T	p.Arg47Cys	Het.	Ⅱb	YES	YES	YES	50	Age > 60y, HTN	IV Heparin	4/5	YES
7	M/30	113	99	39	103	c.456_458delCTT^Δ^	p.phe121del	Het.	Ⅰ	YES	YES	NO	30	NO	Subcutaneous LMWH→Rivaroxaban	5/11	YES
8	M/24	110	105	43	107	c.685 C > T c.938T > C^Δ^	p.Arg197* p.Met281Thr	Comp.Het	Ⅰ	YES	YES	NO	24	NO	MT + CDT→IV Heparin→Rivaroxaban	3/4	YES
9	F/33	102	97	46	135	rs3138521	None	Het.	Ⅰ	YES	YES	NO	33	Preg	Subcutaneous LMWH	7/16	YES
10	M/74	115	120	32	257	c.1346T > A	p.Leu417Gln	Het.	Ⅱc	YES	NO	NO	74	Age > 60y.HTN, Immobil.	Subcutaneous LMWH	4/11	NO
11	F/30	99	87	40	54	c.851T > C^Δ^	p.Met252Thr	Het.	Ⅰ	YES	YES	YES	26	Preg	Subcutaneous LMWH	3/7	YES
12	F/24	107	89	63	324	c.1346T > A^Δ^ c.981 A > G	p.Leu417Gln None	Comp.Het	Ⅱc	NO	NO	NO	NA	recurrent miscarriages	None	2/5	YES
13	F/33	108	93	53	154	c.539G > A	p.Gly180Glu	Het.	Ⅰ	YES	NO	NO	25	Preg, Immobil	None	2/7	NO
14	M/74	95	103	41	269	c.442T > C	p.Ser116Pro	Het.	Ⅱb	YES	YES	NO	74	Age > 60y	Subcutaneous LMWH	NA	NO
15	F/32	105	91	58	254	c.1346T > A	p.Leu417Gln	Het.	Ⅱc	NO	NO	NO	NA	Preg	None	NA	NO
16	M/25	110	119	29	59	c.1 A > G^Δ^ c.1005G > A	p.Tyr2* None	Comp.Het	Ⅰ	YES	YES	NO	25	NO	Subcutaneous LMWH	5/7	YES
17	F/53	89	94	35	104	c.1274G > A	p.Arg393His	Het.	I	YES	YES	YES	50	NO	Subcutaneous LMWH	NA	NO
18	M/37	95	101	46	103	c.462_464delCTT	p.phe122del	Het.	I	YES	YES	YES	32	NO	IV Heparin→Subcutaneous LMWH	5/6	YES
19	M/63	110	92	41	108	c.981 A > G c.1011 A > G	None None	Comp.Het	I	YES	YES	NO	63	Age > 60y, HTN	Subcutaneous LMWH	3/4	NO
20	M/24	116	88.5	50	49	c.964 A > T^Δ^	p.Lys290*	Het.	Ⅰ	YES	YES	NO	24	NO	Subcutaneous LMWH	2/7	NO
21	F/17	88	94	51	102	NA	NA	NA	Ⅰ	YES	NO	NO	17	injury	Subcutaneous LMWH	0/3	NO
22	F/28	102	110	48	36	173,906,489 − 17,391,949^Δ^	NA	Het.	Ⅰ	YES	NO	NO	28	Preg	Subcutaneous LMWH	2/4	NO
23	F/69	98	102	33	256	NA	NA	NA	Ⅱ	YES	YES	NO	69	Age > 60y, injury	IV Heparin→Warfarin	0/4	NO
Reference Range	NA	70–140	80–120	75–125	250–360	NA	NA	NA	NA	NA	NA	NA	NA	NA	NA	NA	NA

“Δ”, Mutation sites first identified by our team; P, proband; G/A, gender/age; M, male; F, female; T, thrombosis, MTh, multiple thrombosis; RT, recurrent thrombosis; C/F, carriers/family members; FHT, family history of thrombosis; Het, heterozygote; Comp.Het, compound heterozygote; Preg, pregnancy; Immobil, immobilization; HTN, hypertension; PIVT, percutaneous intracranial venous thrombectomy; IV heparin, intravenous heparin; LMWH, low-molecular-weight heparin; MT, mechanical thrombectomy; CDT, catheter-directed thrombolysis

NA(Not Available/Not Applicable): In the “Nucleotide change” and “Protein change” columns, NA indicates that no disease-causing mutation was detected. In all other clinical data columns, it indicates that the information is unavailable, unrecorded, or could not be assessed (e.g., in the “C/F” column, NA indicates that family screening was not performed). None: In the “Protein change” column, indicates that the detected genetic mutation does not alter the protein sequence

### Clinical aspects

The majority of probands initially presented with swelling and pain in the lower limbs or headache. No consanguineous marriages were identified during the pedigree investigations of these families. Furthermore, excluding the probands, 44 out of 100 relatives (44%) were confirmed to have the same AT defect. The comprehensive data are available in Supplementary Table 1. All other probands, excluding proband 12 (recurrent miscarriages), and proband 15 (pregnancy termination due to fetal arrest), presented with thrombosis-related symptoms. Most probands presented with DVT, and the remainder with CVST, PE, IVT, or MVT. Multiple and recurrent thromboses were common among them. Typically, the mean age of onset for the first thrombotic event was 30.7 years in Type Ⅰ probands, compared to 57.5 years in Type Ⅱ probands. Notably, more than half of the probands investigated by pedigree analysis had a well-documented family history of thrombosis.

### Molecular characterization

**Fig. 1:**
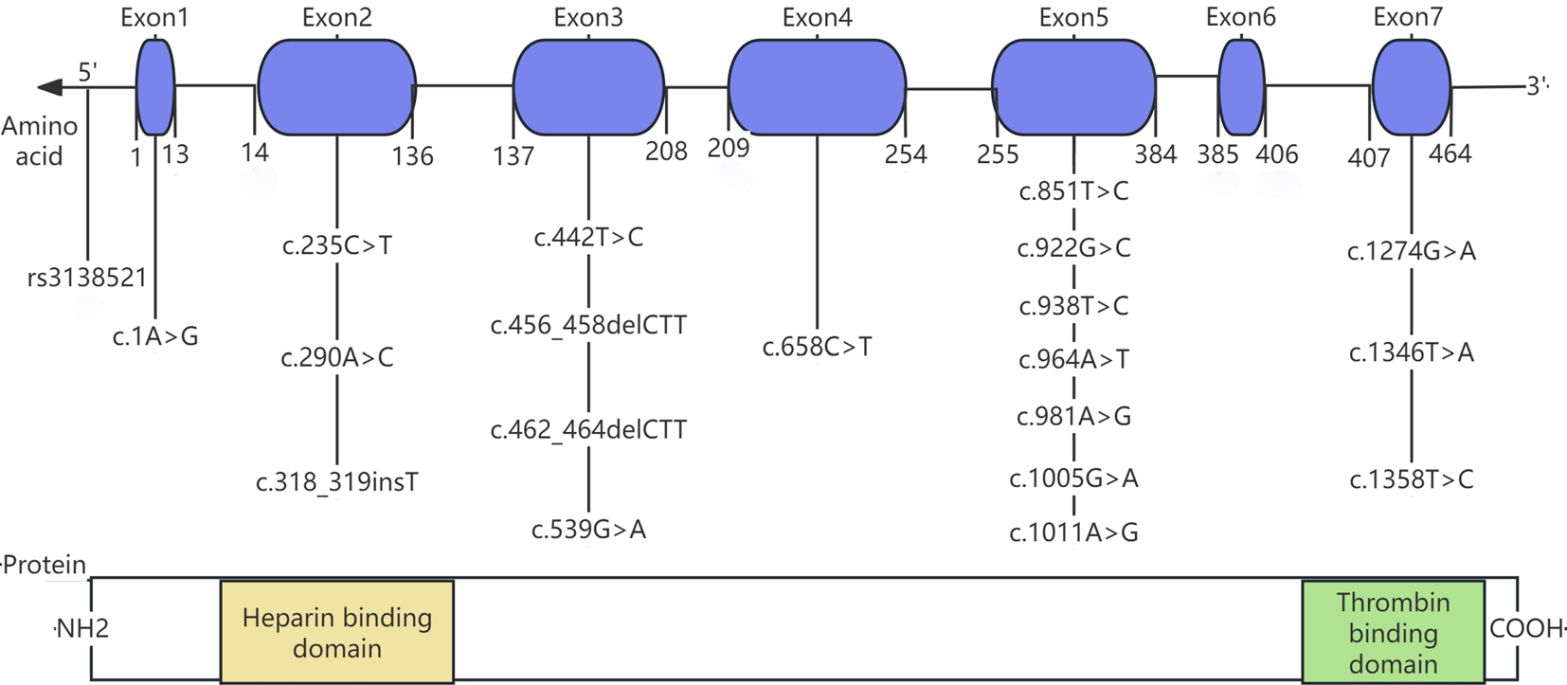
The *SERPINC1* gene and protein domain structures. The *SERPINC1* gene, depicted as a horizontal arrow line, represents genomic DNA. Exons are presented as blue squares and numbered from 1 to 7. The amino acids encoded by each exon are marked below the blue squares and numbered from 1 to 464. The *SERPINC1* protein structure features two major domains: the NH_2_-terminal heparin-binding domain, predominantly encoded by exon 2 and colored yellow, and the COOH-terminal thrombin-binding domain, colored green. The mutations are indicated and arranged below the corresponding exons

**Fig. 2:**
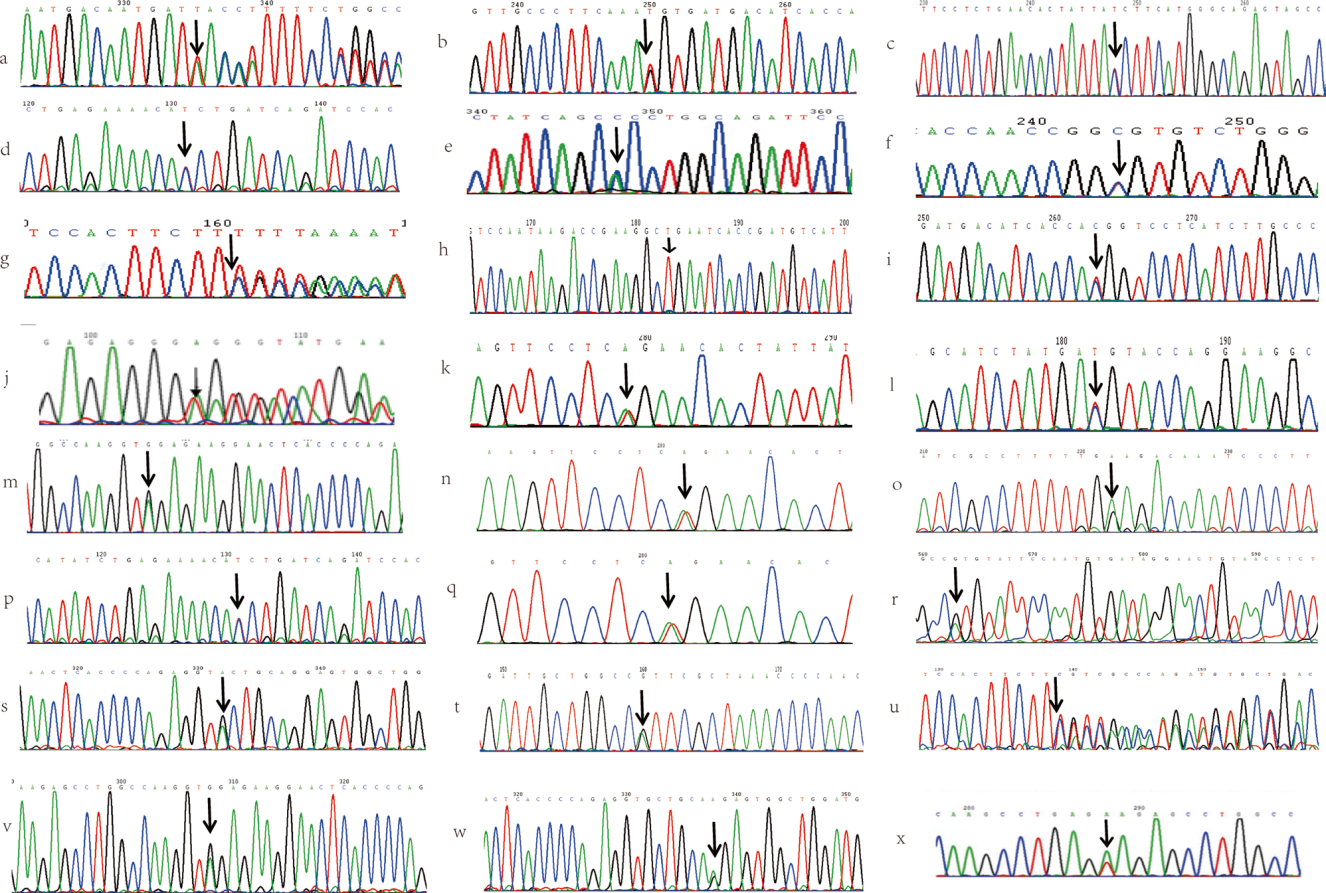
*SERPINC1* gene sequences of all reported mutations in probands, excluding proband 22. The arrow points to the mutant site. (**a**) c.318_319insT mutation in proband 1; (**b**) c.922G > T mutation in proband 1; (**c**) c.1358T > C mutation in proband 2; **d**. c.442T > C mutation in proband 4;** e**. c.290 A > C mutation in proband 5; **f**. c.235 C > T mutation in proband 6; ** g**. c.456_458delCTT mutation in proband 7; **h**. c.685 C > T mutation in proband 8; **i**. c.938T > C mutation in proband 8; ** j**. rs3138521 mutation in proband 9; **k**. c.1346T > A mutation in proband 10; **l**. c.851T > C mutation in proband 11; **m**. c.1346T > A mutation in proband 12; **n**. c.981 A > G mutation in proband 12; **o**. c.539G > A mutation in proband 13; **p**. c.442T > C mutation in proband 14; **q**. c.1346T > A mutation in proband 15; **r**. c.1 A > G mutation in proband 16; **s**. c.1005G > A mutation in proband 16; **t**. c.1274G > A mutation in proband 17; **u**. c.462_464delCTT mutation in proband 18; **v**. c.981 A > G mutation in proband 19; **w**. c.1011 A > G mutation in proband 19; **x**. c.964 A > T mutation in proband 20

**Fig. 3:**
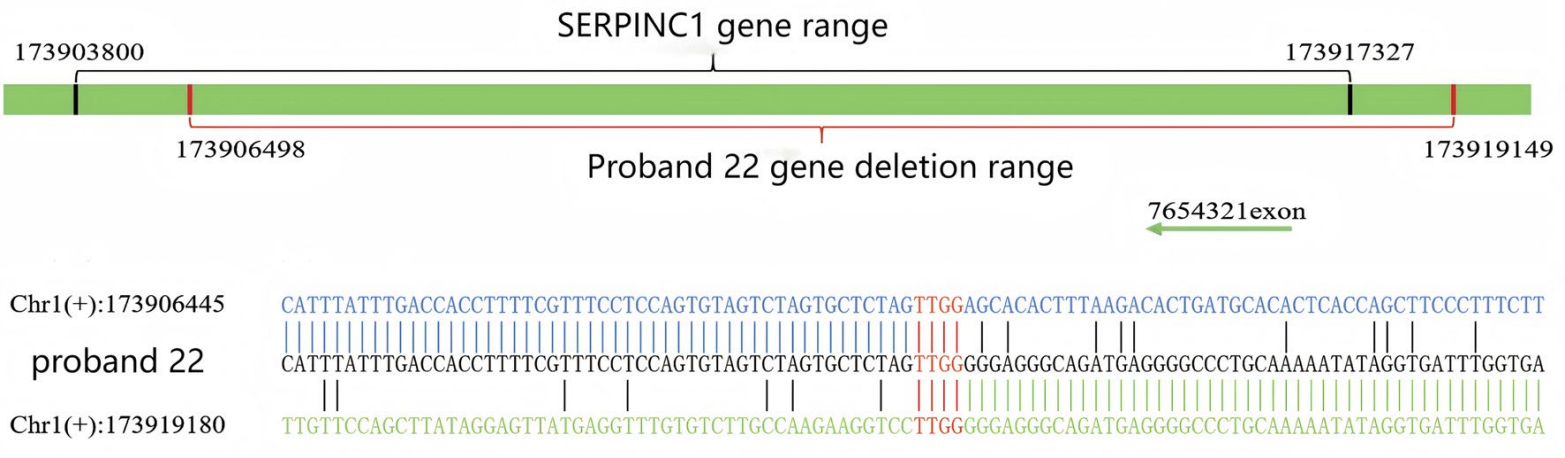
Large-scale deletion of the *SERPINC1* gene in proband 22

Sanger sequencing of the *SERPINC1* gene was performed for all probands to identify potential contributing factors to their thrombosis and to determine the genetic cause underlying the observed reduction in AT activity. Out of 23 probands, 3 (13%) had no detectable mutations, whereas the remaining 20 (87.0%) carried 21 distinct *SERPINC1* mutations. These mutations occurred most frequently in exon 5, followed by exon 3. All 20 probands with mutations were heterozygous: 15 (75%) were simple heterozygotes, and 5 (25%) were compound heterozygotes. Among the 21 identified mutations, 47.6% (10/21) were missense mutations. The cohort exhibited genetic heterogeneity and also included nonsense mutations, deletions, insertions, and single nucleotide polymorphisms (SNPs). The spectrum included nine mutations (e.g., c.318_319insT, c.922G > T, c.456_458delCTT) were initially reported by our team. As shown in Fig. 1, the identified mutations are distributed across the entire *SERPINC1* gene. Figure 2 depicts all probands’ gene sequences except proband 22, who carries a large-scale heterozygous deletion spanning position 173906498 to 173919149 (hg38) in the *SERPINC1* gene, as illustrated in Fig. 3. Compared to the normal range of the *SERPINC1* gene (hg38: 173903800 to 173917327), this constitutes a nearly complete deletion. In addition, proband 9 harbored a rare rs3138521 polymorphism in the 5’ untranslated region (5’ UTR) of the *SERPINC1* gene. Within the promoter region, 345 base pairs (bp) upstream of the transcription start codon, S-type (108 bp) or F-type (32 bp) alleles were identified. The presence of the rs3138521 polymorphism was confirmed by clone sequencing. Proband 16 carried a rare heterozygous c.1 A > G mutation in exon 1, which ablates the start codon, causes a frameshift and results in a truncated protein p.Tyr2*. We identified two recurrent mutations in our cohort: c.1346T > A (p.Leu417Gln) in three probands and c.442T > C (Ser116Pro) in two probands. Overall, the mutation spectrum was diverse, without any race-specific mutations. The detailed data of the probands and their relatives are summarized in Table 1 and supplementary Table 1. All family members tested negative for the prothrombin G20210A mutation and factor V Leiden mutation.

### In silico analysis

**Fig. 4:**
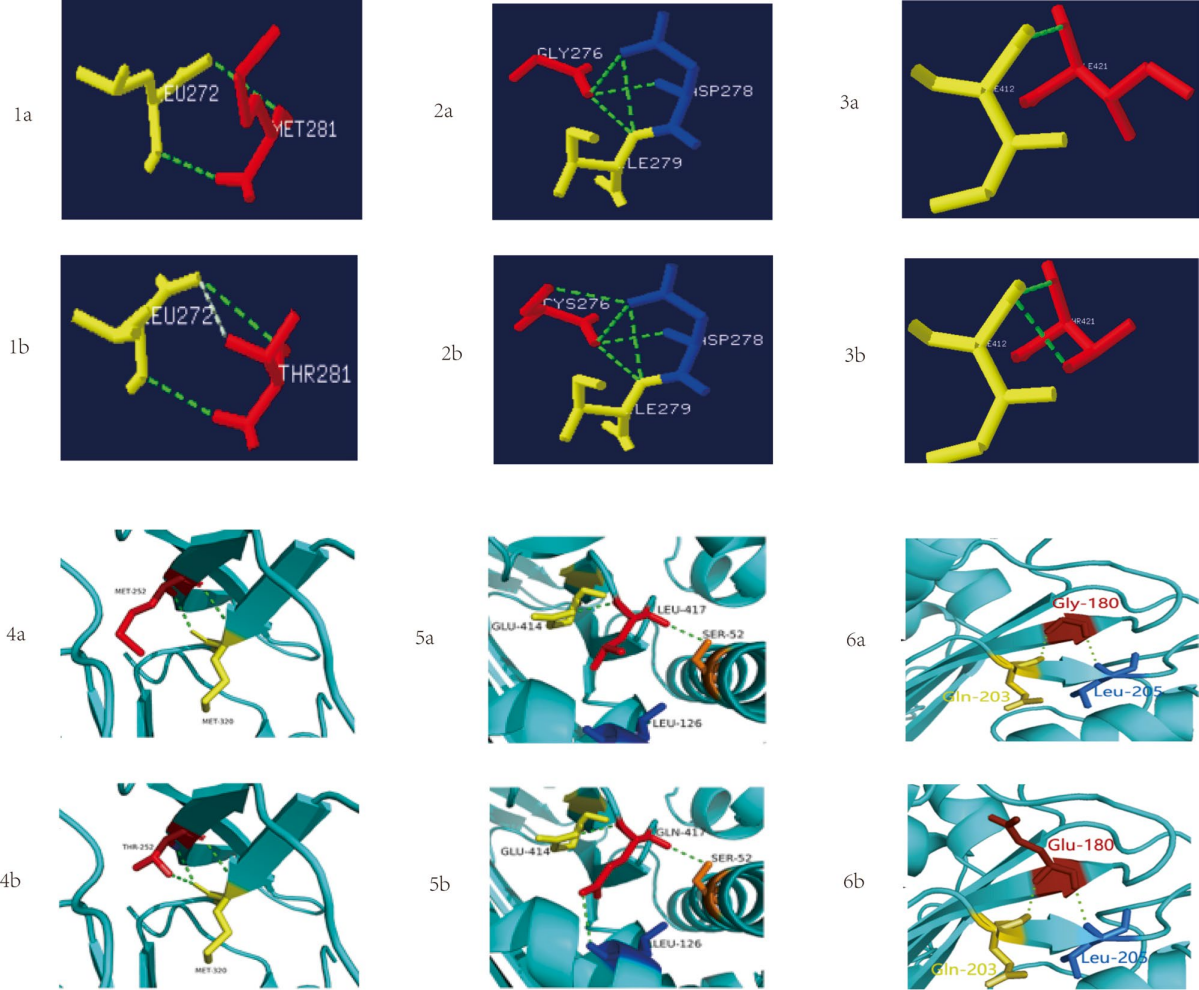
Protein model diagrams of six mutations. **1a** and **1b**: Swiss model of p.Met281Thr mutation: wild type(**1a**); mutant type(**1b**). Red indicates Met281/Thr281; Yellow represents Leu272; Dashed lines denote hydrogen bonds **2a** and **2b**: Swiss model of p.Gly276Cys mutation: wild type(**2a**); mutant type(**2b**). Red indicates Gly276/Cys276; Blue represents Asp278;Yellow represents Ile279; Dashed lines denote hydrogen bonds **3a** and** 3b**: Swiss model of p.Ile421Thr mutation: wild type(**3a**); mutant type(**3b**). Red indicates Ile421/Thr421; Yellow represents Ile412; Dashed lines denote hydrogen bonds **4a** and **4b**: Pymol model of p.Met252Thr mutation: wild type(**4a**); mutant type(**4b**). Red indicates Met252/Thr252; Yellow represents Met320; Dashed lines denote hydrogen bonds **5a** and **5b**: Pymol model of p.Leu417Gln mutation: wild type(**5a**); mutant type(**5b**). Red indicates Leu417/Gln417; Yellow represents Glu414; Orange represents Ser52; Dashed lines denote hydrogen bonds. **6a** and **6b**: Pymol model of p.Gly180Glu mutation: wild type (**6a**); mutant type (**6b**). Red indicates Gly180/Glu180; Yellow represents Gln203; Blue represents Leu205; Dashed lines denote hydrogen bonds. The side-chain of the residue at position 180 extends

Most identified mutations were situated at conserved positions in the *SERPINC1* gene and were predicted to be pathogenic and to impair protein function. The in silico analysis results of the 21 identified mutations are summarized in Table 2. To assess their impact on the AT protein structure, protein modeling was conducted for six mutations (c.938T > C, c.922G > T, c.1358T > C, c.851T > C, c.1346T > A and c.539G > A), as shown in Fig. 4. Protein model analysis of the c.938T > C mutation (Figs. 4-1a, 1b) shows that in the wild-type, Met281 in the S3b region forms two hydrogen bonds with p.Leu272. The substitution of non-polar methionine by polar threonine at this position introduces an additional hydrogen bond, potentially compromising the protein’s structural integrity. Protein model analysis of the c.922G > T mutation (Figs. 4-2a, 2b) revealed that the replacement of non-polar glycine with polar cysteine enables the formation of an additional hydrogen bond between Cys276 and Asp278. This new interaction may affect the local intermolecular structure. Structural modeling shows that the c.1358T > C mutation leads to an isoleucine-to-threonine substitution at residue 421, which facilitates the formation of a new hydrogen bond between Thr421 and Ile412, as depicted in Figs. 4-3a, 3b. Structural analysis of the c.851T > C mutation reveals a change in the hydrogen-bonding network (Fig. 4-4a and 4b). In the wild-type AT model, two hydrogen bonds exist between the main chains of Met252 and Met320. The substitution of methionine with threonine at position 252 replaces a hydrophobic residue with a polar one, which retains the original hydrogen bonds and enables a new one with Met320. Structural analysis of the c.1346T > A mutation (Figs. 4-5a, 5b) shows that the replacement of non-polar leucine with polar glutamine introduces an additional hydrogen bond between Gln417 and Leu126. This may affect the local intermolecular structure integrity. Figure 4-6a depicts the wild-type structure for the c.539G > A locus, where the non-polar, hydrophobic Gly180 forms hydrogen bonds with Gln203 and Leu205. In mutant model (Fig. 4-6b), where Gly180 is replaced by the polar, hydrophilic Glu, these original hydrogen bonds are maintained. However, the changes in amino acid properties, side-chain elongation, and increased molecular weight alter the protein’s spatial conformation.

**Table 2:** The 21 identified mutations and in Silico analysis

Nucleotide change	Protein change	Exon	Type	conservation	PolyPhen-2	PEOVEAN	Mutation Taster	Revel	SIFT	Franklin	PyMOL/SWISS	in vitro expression
c.318_319insT	p.Asn75*	E2	Nonsense	Highly	NA	NA	disease causing/0.999	NA	NA	LP	NO	YES
c.922G > T	p.Gly276Cys	E5	Missense	Highly	0.400	-7.692	disease causing/0.999	0.85	0.001	VUS	YES	YES
c.1358T > C	p.Ile421Thr	E7	Missense	Highly	1.000	-5.480	disease causing/0.999	0.89	0.001	P	YES	YES
c.290 A > C	p.His65Pro	E2	Missense	Highly	0.983	-1.927	disease causing/0.999	0.21	0.150	LP	NO	NO
c.235 C > T	p.Arg47Cys	E2	Missense	Highly	0.986	-2.682	disease causing/0.999	0.74	0.000	P	NO	NO
c.456_458delCTT	p.phe121del	E3	Deletion	Highly	NA	-8.755	disease causing/0.999	NA	NA	P	NO	NO
c.685 C > T	p.Arg197*	E4	Nonsense	Highly	0.998	-5.448	disease causing/0.999	NA	NA	P	NO	NO
c.938T > C	p.Met281Thr	E5	Missense	Highly	0.920	NA	disease causing/0.999	0.96	0.000	P	YES	NO
rs3138521	None	5′UTR	SNP	NA	NA	NA	disease causing/0.997	NA	NA	/	NO	NO
c.1346T > A	p.Leu417Gln	E7	Missense	Highly	1.000	-2.030	disease causing/0.999	0.70	0.061	LP	YES	YES
c.851T > C	p.Met252Thr	E5	Missense	Highly	1.000	-3.150	disease causing/0.999	0.97	0.001	LP	YES	YES
c.539G > A	p.Gly180Glu	E3	Missense	Highly	1.000	-6.890	disease causing/0.999	0.85	0.002	VUS	YES	NO
c.442T > C	p.Ser116Pro	E3	Missense	Highly	0.290	-1.572	disease causing/0.999	0.65	0.200	LP	NO	NO
c.981 A > G	None	E5	SNP	NA	NA	NA	Polymorphism/1.046	NA	NA	B	NO	NO
c.1011 A > G	None	E5	SNP	NA	NA	NA	Polymorphism/0.000	NA	NA	B	NO	NO
c.1 A > G	p.Tyr2*	E1	Nonsense	Highly	1.000	NA	disease causing/0.999	0.6	0.000	VUS	NO	NO
c.1005G > A	None	E5	SNP	NA	NA	NA	disease causing/0.999	NA	NA	B	NO	NO
c.1274G > A	p.Arg393His	E7	Missense	Highly	NA	NA	disease causing/0.999	0.81	0.285	P	NO	NO
c.462_464delCTT	p.phe122del	E3	Deletion	Highly	NA	NA	disease causing/0.999	NA	NA	P	NO	NO
c.964 A > T	p.Lys290*	E5	Nonsense	Highly	NA	NA	disease causing/0.999	NA	NA	LP	NO	YES
173,906,489 − 17,391,949	None	NA	Deletion	NA	NA	NA	NA	NA	NA	NA	NO	NO

NA, Not Available/Not Applicable; None, in the “Protein change” column, indicates no alteration of the protein sequence; PolyPhen-2, scores range from 0.000 (most probably benign) to 1.000 (most probably damaging); PROVEAN, score ≤ − 2.5 (deleterious), >−2.5 (neutral); Mutation Taster, predictions with values closer to 1 are considered more reliable; REVEL, scores range from 0 to 1, with higher scores indicating a greater likelihood of the variant being disease-causing; Franklin, B, benign; LB, likely benign; VUS, variant of uncertain significance; LP, likely pathogenic; P, pathogenic; SIFT, score ≤ 0.05 (damaging ), score > 0.05 (tolerated/acceptable ); SNP, single nucleotide polymorphism

### In vitro expression

In vitro expression and functional characterization were successfully performed for six mutants (p.Asn75*, p.Met252Thr, p.Gly276Cys, p.Lys290*, p.Leu417Gln and p.Ile421Thr). Analysis revealed distinct molecular pathologies underlying the antithrombin deficiency (Table 3). At the transcriptional level, p.Asn75* and p.Gly276Cys exhibited significantly reduced mRNA levels (25.5% and 48.8% of wild-type, respectively), suggesting mechanisms such as nonsense-mediated mRNA decay or transcript destabilization. The other four mutants showed wild-type-like mRNA expression. At the protein level, these defects translated into a spectrum of synthesis and secretion impairments: p.Asn75* and p.Gly276Cys displayed combined defects in both synthesis and secretion; In contrast, only secretion was defective in p.Met252Thr, p.Ile421Thr, and p.Lys290* were impaired primarily in secretion despite robust synthesis;.and p.Leu417Gln showed normal protein synthesis and secretion, indicating a purely functional defect.

**Table 3:** In vitro expression analysis of wild-type and mutant *SERPINC1*

Mutant	RT-qPCR(%)	Conditioned media AT: Ag(%)	Cell lysates AT: Ag(%)
Wild-type	100	100	100
p.Asn75*	25.5	Undetectable	Undetectable
p.Met252Thr	94.6	78.5	149.2
p.Gly276Cys	48.8	41.3	46.9
p.Lys290*	111.5	Undetectable	93.3
p.Leu417Gln	107.6	99.3	109.1
p.Ile421Thr	95.6	77.6	150.3

The expression levels of wild-type and mutant *SERPINC1* in HEK293T cells were analyzed. Values are presented as a percentage of the wild-type

control, which was set to 100% for each assay. ‘Undetectable’ indicates that the antigen level was below the detection limit of the ELISA

## Discussion

ATD is a rare and severe inherited thrombophilia. Antithrombin, with its cofactor heparin enhancing its efficient suicide inhibition, inactivates target proteases, so even mild deficits can notably increase the risk of thrombosis [11, 12]. In these families, inherited ATD (OMIM#613118) [2] in the 21 probands was explained by heterozygoous mutations in the *SERPINC1* gene, which led to reduced AT activity levels of 40%-60%. In total, three probands in this cohort remain unresolved. Two had co-segregating reduced AT in their families, and the remaining one died shortly after presentation, thus no family study was conducted. One potential explanation is that the causative mutation was overlooked due to low sequencing coverage, or that it resides in an unidentified trans-acting gene or a regulatory element of *SERPINC1*. These findings highlight the limitations of the traditional diagnostic approaches and underscore the need for more comprehensive genetic testing strategies, such as whole exome sequencing, whole-genome sequencing, or a combination of multiple complementary techniques. However, numerous reports indicate that about 20–30% of ATD cases exhibit no mutations in *SERPINC1* [13, 14] and may be associated with glycosylation disorders marked by elevated levels of hypoglycosylated antithrombin [15, 16].

The clinical manifestations and severity of ATD vary primarily with the family history of thrombosis as well as the type and location of mutations in the *SERPINC1* gene [17]. Type I ATD patients, mostly heterozygous (homozygosity is rare and possibly life-incompatible), typically carry small deletions, insertions, or single-base substitutions, predisposing them to early-onset thrombosis and a higher recurrence rate [18, 19]. Conversely, type II ATD patients with impaired protein function display a broad clinical spectrum of thrombosis, ranging from mild to life-threatening. In our study, probands with type I defects were more prone to spontaneous thrombosis early in life, whereas those with type II typically developed thrombosis later, following transient triggers. Furthermore, among the type II cases, only subtypes IIb and IIc (not IIa) demonstrated locus-specific mutations in this population. This spectrum of clinical presentations, driven by diverse molecular mechanisms, lies beyond the resolving power of conventional functional assays. Consequently, although functional assays (AT: Ag and AT: A) are the cornerstone for the initial diagnosis and broad classification of ATD, they are often insufficient for a complete diagnosis, making genetic testing an indispensable tool. Indeed, genetic analysis moves beyond confirmation of the hereditary defect to provide authoritative subtyping, which is critical for prognostic stratification and personalized clinical management.

The majority of probands carried predicted deleterious heterozygous mutations, predominantly missense mutations along with other alterations such as insertions, deletions, nonsense mutations, and SNPs. Remarkably, a large-scale deletion and two adjacent small deletions (c.456_458delCTT and c.462_464delCTT) all exhibited a similar AT: A of approximately 40%. This suggests that these deletions might severely impact relevant functions and result in unforeseen consequences. Despite the extreme rarity of documented compound heterozygous ATD cases, our study identified five such probands (cases 1, 8, 12, 16, and 19), a notable subset of the cohort. It can be hypothesized that the reduced AT activity and antigen levels in proband 1 resulted from the combination of c.318_319insT and c.922G > T mutations in *SERPINC1*. The c.922G > T mutation, predicted to alter a conserved amino acid, was of uncertain clinical significance by bioinformatics tools. Proband 1’s mother and maternal grandfather, who carried the c.922G > T mutation, were asymptomatic with normal AT parameters. In contrast, the paternal grandfather, father, aunt, and sister with c.318_319insT mutation showed reduced AT activity and antigen levels; the paternal grandfather and father had a history of DVT. Proband 1 suffered from DVT, CVST and recurrent thrombotic events. The AT protein also contains four glycosylation sites (Asn96, Asn135, Asn155, Asn192) and three disulfide bonds (Cys8-Cys128, Cys21-Cys95, Cys247-Cys430). Alterations in these key residues can affect its structure and function. The c.318_319insT mutation disrupts the reading frame downstream of Asp74, causing a stop codon (UAA) and truncation at Asn75 (Asn75*), which in turn eliminates the glycosylation site at Asn96 and breaks the Cys21-Cys95 disulfide bond. Previous research has shown that p.Cys95Arg breaks the Cys95-Cys21 disulfide bond, impairs antithrombin secretion, causes its retention in the endoplasmic reticulum, and is linked to ATD [20]. Based on this precedent, we hypothesized that this mutation would similarly disrupt both synthesis and secretion, which was subsequently confirmed by our in vitro expression studies. Moreover, protein truncation impairs the N-terminal heparin binding region, diminishing the effectiveness of exogenous heparin, which is consistent with the failed initial heparin therapy in proband 1. Therefore, the patient switched to rivaroxaban for continued anticoagulation. Similar to proband 1, probands 8, 12, and 16 with compound mutations exhibited a marked reduction in AT activity. In contrast, family members with mutations c.938T > C, c.1346T > A, or c.1 A > G had both significantly reduced AT activity and a history of thrombosis. Meanwhile, those carrying mutations c.685 C > T, c.981 A > G, or c.1005G > A exhibited slightly reduced or nearly normal AT activity and remained asymptomatic. We conclude that the mutations c.318_319insT, c.938T > C, c.1346T > A, and c.1 A > G are the main contributors, while other mutations, including c.922G > T, c.685 C > T, and the SNPs c.981 A > G and c.1005G > A, may have synergistic effects. All the aforementioned compound heterozygotes except proband 12 suffered from spontaneous thrombosis. However, most of the other probands with single heterozygous mutations also had thrombi, mostly with identifiable triggers. However, the high incidence of thrombosis may not accurately reflect the link between thrombosis risk and *SERPINC1* mutations in the general population, due to ascertainment bias. Patients are typically tested for thrombophilia only after presenting with thrombotic symptoms or incidentally during prenatal coagulation screening. Analysis of the protein model indicated that mutations can either alter the number or length of hydrogen bonds, thereby impairing the structural integrity and conformation of the protein and affecting AT activity and/or antigen level. We analyzed the impact of six mutations at both the transcriptional and protein levels following in vitro expression. At the transcriptional level, the mutations segregated into two groups: those that reduce mRNA levels through mechanisms like nonsense-mediated decay and those with stable transcript levels. Collectively, the protein-level analyses confirm that the antithrombin deficiency in these mutants arises from distinct molecular mechanisms, encompassing impaired synthesis, defective secretion, or a pure functional deficiency. This indicates that these mutations can significantly affect the AT protein’s biological function and ultimately impact its activity. Proband 19, the sole individual with compound heterozygous polymorphisms (c.981 A > G and c.1011 A > G), developed DVT at 63. Studies have noted that VTE in ATD patients mainly occurs in those under forty [21]. Thus, advanced age and hypertension were likely contributing factors to thrombosis in this proband. Although these polymorphisms do not change the amino acid sequence, they can reduce AT activity and cause VTE [22, 23]. Synonymous mutations can affect protein folding during translation, altering protein structure [24]. Therefore, it is plausible that proband 19’s DVT is associated with these polymorphisms.

In the current study, we also found that a high proportion (72.7%, 8/11) of female probands with ATD were pregnant, and most of these pregnant women developed thrombotic events. It has been reported that even when receiving adequate anticoagulation during pregnancy and the postpartum period, ATD patients remain susceptible to VTE, including fatal thrombotic events [16]. Notably, proband 12 had recurrent miscarriages, and another pregnant proband 15 with the same type IIb mutation (c.1346T > A) experienced fetal arrest. Previous reports [25] indicate that type IIb mutations affecting the heparin-binding site are associated with a lower thrombotic risk, and homozygous cases [26–28] of type IIb or mild type IIc are exceedingly rare. Research on AT-deficient mice and zebrafish [10, 29], along with new human evidence [30], suggests that severe ATD may lead to embryonic lethality, emphasizing the crucial role of antithrombin in embryonic development. Despite carrying the same c.1346T > A mutation as two pregnant women who did not develop thrombosis, a 74-year-old male (proband 10) experienced DVT. Nevertheless, the co-segregation of c.1346T > A with ATD observed in family studies of three probands supports the pathogenic consequences of this mutation. Although our in vitro experiments have proven that type IIb ATD (c.1346T > A) exhibits normal protein synthesis and secretion, the patient’s low AT activity suggests that this mutation may result in dysfunctional AT molecules with impaired anticoagulant activity.

## Conclusions

Our study delineates a spectrum of *SERPINC1* mutations mutations and their associated thrombotic risk, thereby paving the way for refined prognostication. These findings unequivocally establish genetic testing as the cornerstone of precision management in ATD. It is indispensable for confirming diagnosis and heritability, enabling critical subtyping, unmasking compound heterozygosity, and guiding key clinical actions across diverse clinical scenarios, including preventive strategies for women of childbearing age and selection of alternative anticoagulants for patients with heparin-binding site mutations.

## Supplementary Information

Below is the link to the electronic supplementary material.

Supplementary Material 1

## Data Availability

All data generated or analyzed during this study are included in this published article [and its supplementary information files].

## Abbreviations

AT: Antithrombin
ATD: Inherited antithrombin deficiency
PCR: Polymerase chain reaction
HEK293T: Human embryonic kidney 293T cells
RT-qPCR: Quantitative real-time PCR
VTE: Venous thromboembolism
DVT: Deep vein thrombosis
PE: Pulmonary embolism
RCL: Reactive center loop
HGMD: Human gene mutation database
CT: Computed tomography
MRV: Magnetic resonance venography
ED: Emergency department
CVST: Cerebral venous sinus thrombosis
PA: Pulmonary angiography
PPP: Platelet-poor plasma
AT: A: AT activity
AT: Ag: AT antigen
PC: A: plasma protein C activity
PS: A: plasma protein S, activity
SNP: Single nucleotide polymorphism
5' UTR: 5’ untranslated region
bp: Base pair
IVT: Inferior vena cava thrombosis
MVT: Mesenteric vein thrombosis
