# Supplementary material for: SERPINC1 mutations and thrombotic events in inherited antithrombin deficiency: a study on the han population of East China
Source: Orphanet J Rare Dis. 2026 Mar 26;21:108. doi: 10.1186/s13023-026-04200-0 (PMC13020232; doi:10.1186/s13023-026-04200-0)
Supplement: Supplementary file 1 — Supplementary Material 1 [file 13023_2026_4200_MOESM1_ESM.docx]

Supplementary Table 1 Laboratory and Clinical Data of 23 families with inherited Antithrombin Deficiency

| Patient | G/A | Coagulation test results | | | | Nucleotide change | Genotype | Reason for Visit | Thrombus |
| --- | --- | --- | --- | --- | --- | --- | --- | --- | --- |
|  |  | PC:A(%) | PS:A(%) | AT:A(%) | AT:Ag(mg/L) |  |  |  |  |
| **1-Ⅳ1** | **M/21** | **112** | **101** | **43** | **113** | **c.318_319insT，c.922G>T** | **Comp.Het** | **headache** | **DVT,CVST** |
| 1-II1 | F/67 | 127 | 120 | 110 | 293 | Wild | Wild | Family pedigree investigation | NO |
| 1-II2 | M/70 | 112 | 108 | 51 | 134 | c.318_319insT | Het. |  | DVT |
| 1-II3 | M/72 | 95 | 101 | 87 | 215 | c.922G>T | Het. |  | NO |
| 1-II4 | F/68 | 90 | 103 | 109 | 312 | Wild | Wild |  | NO |
| 1-III1 | F/40 | 88 | 95 | 45 | 119 | c.318_319insT | Het. |  | NO |
| 1-III2 | M/47 | 116 | 110 | 50 | 132 | c.318_319insT | Het. |  | DVT |
| 1-III3 | F/46 | 97 | 99 | 88 | 232 | c.922G>T | Het. |  | NO |
| 1-IV2 | F/23 | 92 | 90 | 51 | 135 | c.318_319insT | Het. |  | NO |
| **2-II3** | **F/27** | **119** | **110** | **49** | **52** | **c.1358T>C** | **Het.** | **headache** | **CVST** |
| 2-Ⅰ1 | M/56 | 88 | 76 | 48 | 42 | c.1358T>C | Het. | Family pedigree investigation | NO |
| 2-Ⅰ2 | F/54 | 93 | 87 | 99 | 102 | Wild | Wild |  | NO |
| 2-II1 | F/30 | 98 | 93 | 120 | 118 | Wild | Wild |  | NO |
| 2-II2 | M/28 | 79 | 120 | 107 | 106 | Wild | Wild |  | NO |
| 2-II4 | M/20 | 108 | 120 | 118 | 99 | Wild | Wild |  | NO |
| 2-III1 | F/1 | 124 | 130 | 40 | 45 | c.1358T>C | Het. |  | NO |
| **3** | **F/36** | **71** | **89** | **35** | **45** | **NA** | **NA** | **headache** | **CVST** |
| **4** | **M/59** | **95** | **103** | **46** | **303** | **c.471T>C** | **Het.** | **Lower limb pain** | **DVT,CVST** |
| **5** | **M/34** | **89** | **96** | **66** | **330** | **c.290A>C** | **Het.** | **Lower limb pain,cough** | **DVT,PE** |
| **6-I1** | **M/83** | **81** | **97** | **28** | **298** | **c.235C>T** | **Het.** | **chest pain** | **DVT** |
| 6-II1 | M/57 | 92 | 87 | 46 | 295 | c.235C>T | Het. | Family pedigree investigation | NO |
| 6-II2 | F/53 | 121 | 108 | 102 | 310 | Wild | Wild |  | NO |
| 6-II3 | M/49 | 101 | 93 | 57 | 301 | c.235C>T | Het. |  | NO |
| 6-II4 | F/46 | 89 | 104 | 45 | 287 | c.235C>T | Het. |  | NO |
| **7-IIl2** | **M/30** | **113** | **99** | **39** | **103** | **c.456_458delCTT** | **Het.** | **lower limb pain** | **DVT,PE** |
| 7-II1 | M/66 | 113 | 89 | 123 | 278 | wild | Wild | Family pedigree investigation | NO |
| 7-II2 | F/63 | 106 | 102 | 103 | 289 | wild | Wild |  | NO |
| 7-II3 | M/58 | 115 | 107 | 57 | 114 | c.456_458delCTT | Het. |  | DVT |
| 7-II4 | F/59 | 93 | 102 | 113 | 278 | wild | Wild |  | NO |
| 7-II5 | M/56 | 112 | 114 | 59 | 117 | c.456_458delCTT | Het. |  | DVT |
| 7-IIll | M/36 | 111 | 109 | 44 | 109 | c.456_458delCTT | Het. |  | DVT |
| 7-IIl3 | F/32 | 98 | 101 | 54 | 118 | c.456_458delCTT | Het. |  | NO |
| 7-IIl4 | M/28 | 121 | 115 | 124 | 305 | wild | Wild |  | NO |
| 7-IVl | M/5 | 104 | 105 | 106 | 298 | wild | Wild |  | NO |
| 7-IV2 | M/18 | 121 | 113 | 133 | 299 | wild | Wild |  | NO |
| **8-Ⅱ2** | **M/24** | **110** | **105** | **43** | **107** | **c.685C>T,c.938T>C** | **Comp.Het** | **lower limb pain** | **DVT,IVT** |
| 8-Ⅰ1 | M/52 | 102 | 91 | 50 | 130 | c.938T>C | Het. | Family pedigree investigation | DVT |
| 8-Ⅰ2 | F/50 | 97 | 86 | 110 | 305 | c.685C>T | Het. |  | NO |
| 8-Ⅱ1 | F/27 | 108 | 98 | 105 | 312 | wild | Wild |  | NO |
| **9-Ⅱ6** | **F/33** | **102** | **97** | **46** | **135** | **rs3138521** | **SNP** | **Left lower extremity pain** | **DVT,IVT** |
| 9-Ⅰ1 | F/68 | 95 | 99 | 107 | 280 | wild | Wild | Family pedigree investigation | NO |
| 9-Ⅰ2 | M/62 | 101 | 98 | 40 | 126 | rs3138521 | SNP |  | NO |
| 9-Ⅰ3 | F/58 | 103 | 109 | 104 | 271 | wild | Wild |  | NO |
| 9-Ⅰ4 | F/60 | 101 | 94 | 42 | 132 | rs3138521 | SNP |  | DVT |
| 9-Ⅱ1 | F/45 | 99 | 90 | 43 | 134 | rs3138521 | SNP |  | NO |
| 9-Ⅱ2 | M/44 | 103 | 96 | 113 | 283 | wild | Wild |  | NO |
| 9-Ⅱ3 | F/42 | 100 | 92 | 44 | 138 | rs3138521 | SNP |  | DVT |
| 9-Ⅱ4 | M/39 | 94 | 101 | 99 | 259 | wild | Wild |  | NO |
| 9-Ⅱ5 | M/38 | 91 | 97 | 102 | 281 | wild | Wild |  | NO |
| 9-Ⅱ7 | M/32 | 109 | 111 | 105 | 286 | wild | Wild |  | NO |
| 9-Ⅱ8 | M/29 | 103 | 94 | 44 | 132 | rs3138521 | SNP |  | NO |
| 9-Ⅱ9 | F/27 | 98 | 89 | 108 | 290 | wild | Wild |  | NO |
| 9-Ⅲ1 | M/17 | 105 | 94 | 96 | 276 | wild | Wild |  | NO |
| 9-Ⅲ2 | M/7 | 103 | 98 | 112 | 347 | wild | Wild |  | NO |
| 9-Ⅲ3 | M/5 | 99 | 96 | 50 | 145 | rs3138521 | SNP |  | NO |
| **10-I1** | **M/74** | **115** | **120** | **32** | **257** | **c.1346T>A** | **Het.** | **left-sided limb weakness** | **DVT** |
| 10-Ⅰ2 | F/70 | 90 | 86 | 109 | 262 | wild | Wild | Family pedigree investigation | NO |
| 10-Ⅱ1 | F/50 | 89 | 98 | 110 | 270 | wild | Wild |  | NO |
| 10-Ⅱ2 | M/45 | 109 | 105 | 43 | 255 | c.1346T>A | Het. |  | NO |
| 10-Ⅱ3 | F/44 | 86 | 70 | 109 | 281 | wild | Wild |  | NO |
| 10-Ⅱ4 | F/43 | 94 | 85 | 52 | 264 | c.1346T>A | Het. |  | NO |
| 10-Ⅱ5 | M/45 | 105 | 120 | 117 | 278 | wild | Wild |  | NO |
| 10-Ⅱ6 | M/40 | 127 | 130 | 97 | 290 | wild | Wild |  | NO |
| 10-Ⅲ1 | M/22 | 106 | 96 | 106 | 288 | wild | Wild |  | NO |
| 10-Ⅲ2 | M/21 | 99 | 87 | 114 | 279 | wild | Wild |  | NO |
| 10-Ⅲ3 | F/18 | 87 | 102 | 48 | 274 | c.1346T>A | Het. |  | NO |
| **11-Ⅲ2** | **F/30** | **99** | **87** | **40** | **54** | **c.851T>C*** | **Het.** | **lower limb pain** | **IVT,PE** |
| 11-Ⅰ2 | F/86 | 90 | 86 | 109 | 102 | wild | Wild | Family pedigree investigation | NO |
| 11-Ⅱ1 | M/54 | 89 | 98 | 38 | 44 | c.851T>C | Het. |  | DVT |
| 11-Ⅱ2 | M/53 | 109 | 105 | 108 | 109 | wild | Wild |  | NO |
| 11-Ⅱ3 | F/51 | 86 | 70 | 50 | 48 | c.851T>C | Het. |  | DVT |
| 11-Ⅲ1 | M/32 | 106 | 96 | 106 | 113 | wild | Wild |  | NO |
| 11-Ⅳ1 | F/4 | 87 | 102 | 110 | 94 | wild | Wild |  | NO |
| **12-Ⅱ2** | **F/24** | **107** | **89** | **63** | **324** | **c.1346T>A,c.981A>G** | **Comp.Het** | **Fetal demise** | **NO** |
| 12-Ⅰ2 | F/50 | 101 | 94 | 102 | 309 | Wild | Wild | Family pedigree investigation | NO |
| 12-Ⅱ2 | F/26 | 97 | 91 | 106 | 294 | Wild | Wild |  | NO |
| 12-Ⅱ3 | M/28 | 109 | 103 | 100 | 270 | Wild | Wild |  | NO |
| 12-Ⅲ1 | F/4 | 96 | 87 | 58 | 330 | c.1346T>A | Het. |  | NO |
| **13-Ⅲ2** | **F/33** | **108** | **93** | **53** | **154** | **c.538G>A** | **Het.** | **spontaneous abortions** | **DVT** |
| 13-Ⅱ1 | F/55 | 102 | 87 | 102 | 318 | wild | Wild | Family pedigree investigation | NO |
| 13-Ⅱ2 | M/56 | 96 | 98 | 51 | 148 | c.539G>A | Het. |  | NO |
| 13-Ⅲ1 | M/34 | 100 | 105 | 105 | 297 | wild | Wild |  | NO |
| 13-Ⅲ3 | F/31 | 89 | 94 | 112 | 298 | wild | Wild |  | NO |
| 13-Ⅲ4 | M/33 | 92 | 89 | 108 | 319 | wild | Wild |  | NO |
| 13-Ⅳ1 | M/5 | 91 | 108 | 107 | 321 | wild | Wild |  | NO |
| **14** | **M/74** | **95** | **103** | **44** | **269** | **c.442T>C** | **Het.** | **lower limb weakness** | **IVT,DVT** |
| **15** | **F/32** | **105** | **91** | **58** | **254** | **c.1346T>A** | **Het.** | **Fetal demise** | **NO** |
| **16-III1** | **M/25** | **110** | **119** | **29** | **59** | **c.1A>G,c.1005G>A** | **Comp.Het** | **lower limb swelling** | **IVT,DVT,PE** |
| 16-I2 | F/71 | 88 | 76 | `100 | 303 | wild | Wild | Family pedigree investigation | NO |
| 16-I3 | M/70 | 93 | 87 | 99 | 293 | wild | Wild |  | NO |
| 16-I4 | F/70 | 98 | 93 | 86 | 200 | c.1005G>A | SNP |  | NO |
| 16-II1 | M/51 | 124 | 130 | 65 | 99 | c.1A>G | Het. |  | DVT |
| 16-II2 | F/49 | 79 | 120 | 90 | 206 | c.1005G>A | SNP |  | NO |
| 16-III2 | M/23 | 108 | 120 | 83 | 180 | c.1005G>A | SNP |  | NO |
| **17** | **F/53** | **89** | **94** | **35** | **104** | **c.1274G>A** | **Het.** | **abdominal pain** | **MVT,DVT** |
| **18-III1** | **M/37** | **95** | **101** | **46** | **103** | **c.462_464delCTT** | **Het.** | **lower limb swelling and pain** | **PE** |
| 18-II1 | M/62 | 99 | 103 | 88 | 213 | wild | Wild | Family pedigree investigation | NO |
| 18-II2 | F/60 | 101 | 97 | 55 | 111 | c.462_464delCTT | Het. |  | NO |
| 18-II3 | M/58 | 89 | 93 | 50 | 108 | c.462_464delCTT | Het. |  | DVT |
| 18-II4 | M/56 | 98 | 88 | 44 | 111 | c.462_464delCTT | Het. |  | DVT |
| 18-II5 | F/53 | 94 | 103 | 49 | 105 | c.462_464delCTT | Het. |  | DVT |
| **19-II1** | **M/63** | **110** | **92** | **41** | **108** | **c.981A>G,c.1011A>G** | **Comp.Het** | **lower limb swelling and pain** | **IVT,DVT** |
| 19-II2 | F/61 | 109 | 105 | 108 | 242 | Wild | Wild | Family pedigree investigation | NO |
| 19-III1 | M/34 | 106 | 96 | 76 | 188 | c.981A>G | SNP |  | NO |
| 19-III2 | F/32 | 99 | 87 | 72 | 194 | c.1011A>G | SNP |  | NO |
| **20-III1** | **M/24** | **116** | **88.5** | **50** | **49** | **c.964A>T*** | **Het.** | **chest tightness** | **IVT,DVT,PE** |
| 20-I1 | M/76 | 105 | 97 | 102 | 100 | Wild | Wild | Family pedigree investigation | NO |
| 20-II1 | F/48 | 95 | 89 | 98 | 96 | Wild | Wild |  | NO |
| 20-II2 | F/46 | 110 | 92 | 44 | 46 | c.964A>T | Het. |  | NO |
| 20-II3 | M/47 | 120 | 102 | 115 | 114 | Wild | Wild |  | NO |
| 20-II4 | M/43 | 107 | 88 | 108 | 107 | Wild | Wild |  | NO |
| 20-III2 | F/23 | 114 | 93 | 105 | 106 | Wild | Wild |  | NO |
| **21-II1** | **F/17** | **88** | **94** | **51** | **102** | **NA** | **NA** | **fall-related injury** | **DVT** |
| 21-I1 | M/41 | 99 | 87 | 50 | 110 | NA | NA | Family pedigree investigation | NO |
| 21-I2 | F/40 | 93 | 101 | 111 | 266 | NA | NA |  | NO |
| **22-II1** | **F/28** | **102** | **110** | **48** | **36** | **173906489-17391949** | **Het.** | **chest tightness** | **PE** |
| 22-I1 | F/47 | 87 | 95 | 44 | 36 | 173906489-17391949 | Het. | Family pedigree investigation | NO |
| 22-I2 | M/50 | 105 | 97 | 103 | 210 | Wild | Wild |  | NO |
| 22-II2 | M/29 | 95 | 89 | 105 | 265 | Wild | Wild |  | NO |
| **23-I1** | **F/69** | **120** | **102** | **33** | **256** | **NA** | **NA** | **lower back pain** | **PE,DVT** |
| 23-II1 | M/44 | 107 | 88 | 49 | 261 | NA | NA | Family pedigree investigation | NO |
| 23-II2 | F/41 | 114 | 93 | 107 | 285 | NA | NA |  | NO |
| 23-III1 | F/11 | 88 | 94 | 110 | 273 | NA | NA |  | NO |
| Reference  Range | NA | 70-140 | 80-120 | 75-125 | 250-360 | NA | NA | NA | NA |

Probands are indicated in bold. NA, Not Available/Not Applicable; in the "Nucleotide change" columns, NA indicates that no disease-causing mutation was detected. G/A, gender/age; M, male; F, female; Het, heterozygote; Comp.Het,

compound heterozygote; SNP, single nucleotide polymorphism; DVT, deep vein thrombosis; CVST, cerebral venous sinus thrombosis; PE, pulmonary embolism; IVT, inferior vena cava thrombosis; MVT, mesenteric venous thrombosis.
